# Circulating Tumor Cell Clusters Are Frequently Detected in Women with Early-Stage Breast Cancer

**DOI:** 10.3390/cancers13102356

**Published:** 2021-05-13

**Authors:** Carolina Reduzzi, Serena Di Cosimo, Lorenzo Gerratana, Rosita Motta, Antonia Martinetti, Andrea Vingiani, Paolo D’Amico, Youbin Zhang, Marta Vismara, Catherine Depretto, Gianfranco Scaperrotta, Secondo Folli, Giancarlo Pruneri, Massimo Cristofanilli, Maria Grazia Daidone, Vera Cappelletti

**Affiliations:** 1 Department of Applied Research and Technological Development, Fondazione IRCCS Istituto Nazionale dei Tumori di Milano, 20133 Milan, Italy; carolina.reduzzi@northwestern.edu (C.R.); serena.dicosimo@istitutotumori.mi.it (S.D.C.); rosita.motta@istitutotumori.mi.it (R.M.); marta.vismara@istitutotumori.mi.it (M.V.); mariagrazia.daidone@istitutotumori.mi.it (M.G.D.); 2 Robert H. Lurie Comprehensive Cancer Center, Department of Medicine-Hematology and Oncology, Feinberg School of Medicine, Northwestern University, Chicago, IL 60611, USA; lorenzo.gerratana@northwestern.edu (L.G.); paolo.damico@northwestern.edu (P.D.); youbin.zhang1@northwestern.edu (Y.Z.); Massimo.cristofanilli@nm.org (M.C.); 3 Department of Medicine (DAME), University of Udine, 33100 Udine, Italy; 4 Department of Medical Oncology, Centro di Riferimento Oncologico di Aviano (CRO), IRCCS, 33081 Aviano, Italy; 5 Medical Oncology Unit, Fondazione IRCCS Istituto Nazionale dei Tumori, 20133 Milan, Italy; Antonia.Martinetti@istitutotumori.mi.it; 6 Department of Pathology and Laboratory Medicine, Fondazione IRCCS Istituto Nazionale dei Tumori di Milano, Via Giacomo Venezian 1, 20133 Milan, Italy; Andrea.Vingiani@istitutotumori.mi.it (A.V.); Giancarlo.Pruneri@istitutotumori.mi.it (G.P.); 7 Oncology and Hemato-Oncology Department, University of Milan, Via Festa del Perdono 7, 20122 Milano, Italy; 8 New Drugs and Early Drug Development for Innovative Therapies Division, IEO, European Institute of Oncology IRCCS, 20133 Milan, Italy; 9 Department of Radiology, Fondazione IRCCS Istituto Nazionale dei Tumori di Milano, 20133 Milan, Italy; Catherine.Depretto@istitutotumori.mi.it (C.D.); Gianfranco.Scaperrotta@istitutotumori.mi.it (G.S.); 10 Breast Cancer Unit, Fondazione IRCCS Istituto Nazionale dei Tumori di Milano, 20133 Milan, Italy; Secondo.Folli@istitutotumori.mi.it

**Keywords:** circulating tumor cell clusters, liquid biopsy, early breast cancer, metastatic breast cancer, circulating tumor microemboli, size-based enrichment

## Abstract

**Simple Summary:**

Metastases cause the majority of breast cancer-related deaths. Circulating tumor cells (CTCs), and in particular CTC-clusters, are considered the seeds of metastasis, but their analysis in the early-stages of the disease has so far been limited by the fact that, by using conventional and epithelial-based technologies (as the FDA-approved CellSearch platform), they are more often detected in the metastatic setting. It is known, however, that cancer cells are heterogeneous and can downregulate the expression of epithelial markers, thus limiting the detection capability of epithelial-based technologies. Here, we show that it is possible to increase CTC-cluster detection by using an epithope-independent technology based on blood filtration, and in particular that this strategy allows to detect a high number of CTC-clusters in stage II-III breast cancer patients, before and during neoadjuvant treatment. Our results therefore offer a new opportunity to deepen our understanding of the cancer dissemination process in its early steps.

**Abstract:**

The clinical relevance of circulating tumor cell clusters (CTC-clusters) in breast cancer (BC) has been mostly studied using the CellSearch^®^, a marker-dependent method detecting only epithelial-enriched clusters. However, due to epithelial-to-mesenchymal transition, resorting to marker-independent approaches can improve CTC-cluster detection. Blood samples collected from healthy donors and spiked-in with tumor mammospheres, or from BC patients, were processed for CTC-cluster detection with 3 technologies: CellSearch^®^, CellSieve™ filters, and ScreenCell^®^ filters. In spiked-in samples, the 3 technologies showed similar recovery capability, whereas, in 19 clinical samples processed in parallel with CellSearch^®^ and CellSieve™ filters, filtration allowed us to detect more CTC-clusters than CellSearch^®^ (median number = 7 versus 1, *p* = 0.0038). Next, samples from 37 early BC (EBC) and 23 metastatic BC (MBC) patients were processed using ScreenCell^®^ filters for attaining both unbiased enrichment and marker-independent identification (based on cytomorphological criteria). At baseline, CTC-clusters were detected in 70% of EBC cases and in 20% of MBC patients (median number = 2, range 0–20, versus 0, range 0–15, *p* = 0.0015). Marker-independent approaches for CTC-cluster assessment improve detection and show that CTC-clusters are more frequent in EBC than in MBC patients, a novel finding suggesting that dissemination of CTC-clusters is an early event in BC natural history.

## 1. Introduction

Metastatic spreading is the main cause for death in patients diagnosed with cancer. This process is promoted in its initial steps by cancer cells released from the primary tumor into the blood stream. Accordingly, a large amount of data has been collected across different tumor types linking the dissemination of circulating tumor cells (CTCs) with both poor prognosis and treatment failure/resistance [1].

Nonetheless, single CTCs are inefficient in sustaining metastatic dissemination as, to be able to colonize new sites, they must overcome numerous obstacles such as avoid anoikis, escape immunological control by circulating immune cells, and resist sharing stress due to fluid circulation, resulting in the fact that most CTCs do not survive long in the circulation [2,3]. Therefore, being able to interact with other CTCs or with other cells by generating homo- or heterotypic CTC-clusters appears a biologically reasonable solution for increasing the metastatic potential of CTCs once they are facing the hostile blood environment.

Functional studies employing animal models and patient-derived data [4,5,6,7] support a role of CTC-clusters in tumor dissemination and metastasis formation in breast cancer (BC). Such studies also offer hints on the biology of clusters revealing the mechanistic basis for their association with poor outcome and suggesting possible targets for specific treatments aiming at interfering with CTC-clusters formation and metastatic dissemination.

It is well known that metastatic dissemination occurs at early stages and is followed by a prolonged dormant status of these early disseminated cells [8,9,10]. This observation is supported by data demonstrating that enumeration of single CTCs predicts progression-free survival and overall survival also in early breast cancer (EBC) patients (women with no evidence for distant metastases), both prior [11,12] or after [13] breast surgery. Therefore, addressing the presence of CTC-clusters in BC patients without clinically overt metastases holds promise to gain important hints about the dissemination process.

However, this issue has not yet been addressed and, in BC, most studies evaluating the clinical relevance of CTC-clusters have been limited to patients with metastatic or advanced disease [14,15,16,17,18,19,20]. Overall, these studies suggest a direct association between detection of CTC-clusters and poor clinical outcome, although the heterogeneous patient case series, technical issues in CTC-cluster enumeration and variable definitions of CTC-clusters must be taken into account as possible limitations and confounding factors.

Notably, all the mentioned studies used the CellSearch^®^ for CTC-cluster detection, which is possibly not the ideal method for CTC-cluster identification. The CellSearch^®^ is a platform specifically developed for assuring high detection of single CTCs with epithelial features and for attaining standardization of their enumeration [21]. No data are available on its performance for CTC-cluster detection both in terms of recovery and of the integrity of isolated clusters. The CellSearch^®^ approach includes a CTC-enrichment step employing ferrofluid nanoparticles with antibodies targeting EpCAM, which operates a selection in favor of clusters with exquisite epithelial features and possibly excludes larger CTC-clusters [22], which could result in an underestimation in CTC-cluster enumeration. Moreover, epithelial-to-mesenchymal transition (EMT) is recognized as an important driver of tumor invasion and metastatic dissemination [23], and literature data supported an increasing detection of mesenchymal markers in CTC-clusters compared to single CTCs in breast cancer patients [24].

Recent studies by our group [25] and by an independent laboratory [26] reported the detection of CTC-clusters in small cohorts of early-stage BC patients and confirmed their malignancy by genomic profiling of individually isolated clusters. In particular, thanks to the use of a blood-filtration technology, we were able to detect CTC-clusters in 6/6 EBC patient samples and assessed the presence of DNA aberrations in 96% of 48 analyzed clusters [25]. These results suggest that CTC-clusters are frequent in EBC but are not detected by the CellSearch. Thus, investigating the use of epitope-independent methods, compared to the CellSearch^®^, for CTC-cluster detection, is urgently needed to be able to fully appraise the actual clinical value of CTC-cluster in all BC stages.

Here, we hypothesized that resorting to epitope-independent approaches, such as blood-filtration, can increase the detection of CTC-clusters, and that, by using these approaches, it is possible to identify CTC-clusters in EBC patients. To test our hypothesis we first compared, in a series of spiked-in and clinical samples, the number of CTC-clusters recovered using the CellSearch^®^ platform and two size-exclusion methods based on a short-time filtration that allows for the detection of both epithelial and non-epithelial CTC-clusters. Thereafter, we implemented the recovery of CTC-clusters by filtration in a prospective study involving patients with both EBC and metastatic BC (MBC) to analyze CTC-cluster detection with respect to patient and primary tumor features.

## 2. Results

### 2.1. Comparison of Different Strategies for CTC-Cluster Identification

#### 2.1.1. Technical Validation of Approaches used for CTC-Cluster Detection

To explore technical limitations of standard (CellSearch^®^) and filtration-based methods for CTC-cluster detection, spiking experiments were performed comparing size-exclusion approaches with the CellSearch^®^ method (the currently most frequently used method in CTC-cluster studies). In particular, we compared three technologies: the CellSearch^®^, the CellSieve™ filters and the ScreenCell^®^ filters. The latter two are very similar for the enrichment strategy (based on short-time filtration through a membrane with pores of 7 and 6.5 µm, respectively), but differ for the criteria employed for the identification of tumor cells. CellSieve™ filters include an identification based on marker expression similar to that of CellSearch^®^ (CK^pos^ and CD45^neg^ cells), whereas ScreenCell^®^ filters’ identification is based on cytomorphological evaluation.

Mammospheres derived from the MCF7 breast cancer cell line were used as surrogates of CTC-clusters. For each technology, 10 mammospheres were spiked into healthy donor blood samples (*n* = 8), and subsequently processed for CTC-cluster enrichment. For ScreenCell^®^ only, two spiking experiments were performed using PBS supplemented with HSA, instead of blood; this was done to test the stress associated with the filtration process itself, since, for this technology only, fresh blood is used for the spiking step and the presence of active immune cells from the donor might have an impact on mammospheres (for CellSearch^®^ and CellSieve™ the blood is instead collected in CellSave tubes containing a preservative which fix blood cells). The mammosphere recovery rates for each of the 10 spiking experiments are reported in Table 1. 

All three technologies showed similar recovery ranging from 60% to 100%. The impossibility of recovering 100% of the mammospheres in each sample suggests that a partial dissociation of the mammospheres occurred, as also supported by the presence of single tumor cells and fragments of mammospheres in the samples. However, disruption was not specifically induced by filtration; in fact all the samples with a 100% recovery were processed with filters.

Another possible concern about using filtration devices for CTC-cluster identification is the possibility of the formation of aggregates on the filtration membrane during the processing, resulting in the identification of fictitious CTC-clusters. To exclude this possibility, we spiked single MCF7 cells in three healthy donor blood samples (*n* = 30 MCF7 cells per sample). The samples were processed with CellSieve™ filters and with ScreenCell^®^ filters (2 and 1 sample, respectively). We observed the presence of only 1 aggregate of two tumor cells, on one CellSieve™ filter, indicating that filtration does not induce the formation of artifactual clusters.

#### 2.1.2. CTC-Cluster Detection in Clinical Samples Using an Epithelial-Based and a Size-Based Approach

**Figure 1:**
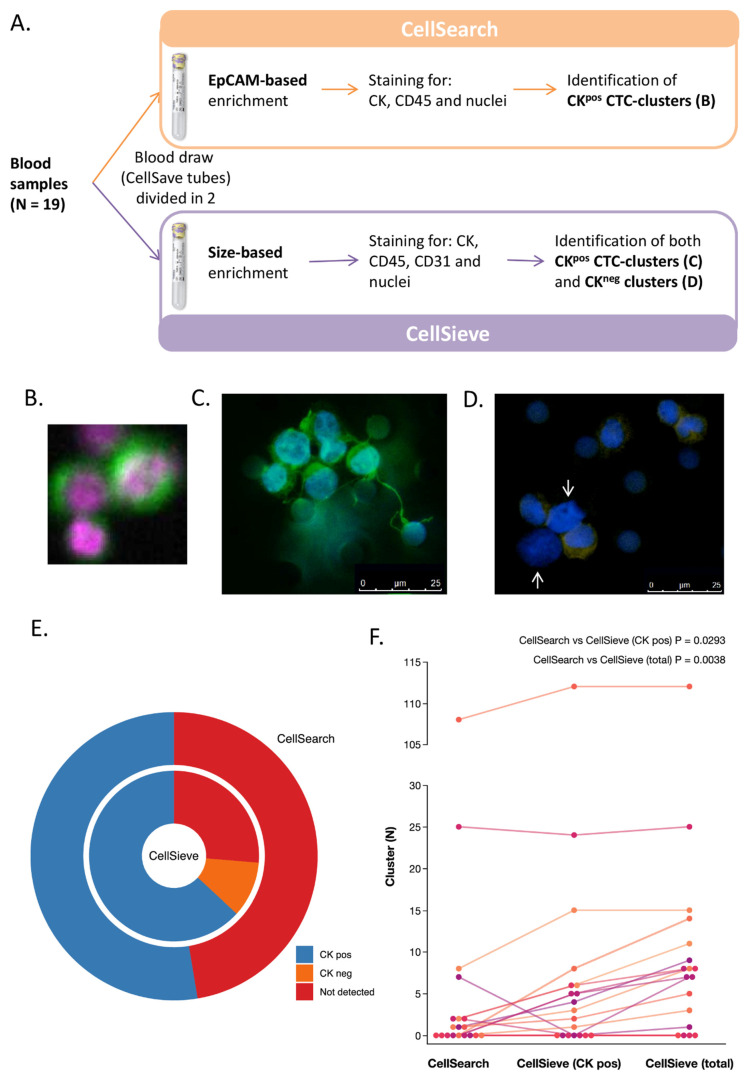
Comparison of CellSearch and CellSieve for CTC-cluster detection in clinical samples. (**A**) Nineteen blood samples collected from patients with MBC were processed in parallel with CellSearch and CellSieve for the detection of CTC-clusters. (**B**) Representative image of a CK^pos^ CTC-cluster detected by CellSearch (green = CK; pink = DAPI; 10× magnification). (**C**,**D**) Representative images of a CK^pos^ (C) and a CK^neg^ (D) cluster detected by CellSieve (green = CK; blue = DAPI; yellow = CD45; the white arrows indicate 2 CD45^neg^/CK^neg^ cells inside the cluster). (**E**) Doughnut plot showing the percentages of samples containing CK^pos^ CTC-clusters (blue) analyzed by CellSearch (outer circle, 53%) and CellSieve (inner circle, 79%). Positivity threshold was set at 1 CTC-cluster/7.5 mL of blood. The percentage of CellSieve samples containing only CK^neg^ clusters are shown in orange (5%). (**F**) Spaghetti plot showing the numbers of CTC-clusters detected in each sample analyzed by CellSearch and CellSieve. For CellSieve samples, both the counts of CK^pos^ CTC-clusters only and of CK^pos^ plus CK^neg^ clusters (CellSieve total) are reported (colors are arbitrary assigned for increasing readability of the graph only).

Once we assessed that the ability of enriching clusters for the three technologies was similar, we next aimed at evaluating whether phenotypic heterogeneity of CTC-clusters in clinical samples (i.e., the presence of both epithelial and non-epithelial clusters) could have an impact on CTC-cluster detection by the epithelial marker-based CellSearch^®^ platform, compared to the marker-independent and size-based approaches. In that respect, 19 blood samples collected from 16 patients with MBC were processed in parallel with CellSearch^®^ and CellSieve™ filters (Figure 1A). For this analysis, CellSieve™ was used as the representative among the two filtration methods, since its enrichment strategy is the same of ScreenCell filters (based on size), but its identification criteria are based on the detection of epithelial markers, and therefore allow for the distinction between epithelial and non-epithelial clusters (not possible with ScreenCell^®^ filters).

Blood samples were collected from clinically selected patients with highly aggressive disease and during disease progression to increase the probability of CTC-cluster presence (patients’ clinico-pathological characteristics are reported in Table S1). For samples processed with the CellSearch^®^, only CTC-clusters expressing CK (CK^pos^ CTC-clusters, defined as groups of two or more cells showing CK^pos^ and CD45^neg^ staining, Figure 1B) could be detected, whereas for samples processed with CellSieve™ filters it was possible to identify both CK^pos^ and CK^neg^ clusters (Figure 1C,D, respectively). CK^neg^ clusters were defined as groups of two or more cells showing a CK^neg^, CD45^neg^ and CD31^neg^ staining (the latter marker allowing for the exclusion of endothelial cell clusters). CD31 expression was unexpectedly observed also in a few CK^pos^ CTC-clusters (Figure S1). These clusters, CK^pos^ and CD45^neg^, were included in the analysis.

We detected ≥1 CK^pos^ CTC-clusters in 10 samples by using the CellSearch^®^ and in 15 samples by using CellSieve™ filters (Table S2). Moreover, in the samples processed by filtration, CK^neg^ clusters were observed in 12 out of 18 evaluable samples, in 1 case alone and in 11 cases together with CK^pos^ CTC-clusters.

By adopting as positivity threshold the presence of one single cluster per sample, we observed an increase in positivity rates from 53% in CellSearch^®^ samples, to 79% and 84% in CellSieve™ samples when considering only CK^pos^ or CK^pos^ and CK^neg^ clusters, respectively (Figure 1E). Moreover, the absolute numbers of detected clusters were higher in samples processed with CellSieve™ filters than with the CellSearch^®^ (Figure 1F; Table S2). In samples processed with the CellSearch^®^, a median of 1 CK^pos^ CTC-cluster (interquartile range, IQR = 0–2; range 0–108) was identified, compared to a median of 3 CK^pos^ CTC-clusters (IQR 1–6; range 0–112) for samples processed with CellSieve™ filters (*p* = 0.0293). The increase in cluster counts for samples processed with CellSieve™ filters was even higher when considering CK^pos^ and CK^neg^ clusters together (median = 7, IQR 1–11; range 0–112, *p* = 0.0038).

These results suggest that by using a size-based and marker-independent approach it is possible to detect a higher number of clusters, allowing them to also be identified in patients considered CTC-cluster negative by the CellSearch^®^. However, the observed phenotypic heterogeneity of clusters in BC patient samples, and in particular the presence of CK^neg^ clusters, highlighted an important limitation of CellSieve™ technology, which was able to enrich this type of clusters, but did not allow for reliable assessment of their malignancy (since they were only DAPI^pos^). On the other hand, ScreenCell^®^ technology had the same ability of enriching CK^neg^ clusters (since it is size-based as well), but its identification was based on cytomorphological evaluation and was therefore not dependent on the expression of any specific tumor marker. Moreover, we recently demonstrated the technical validity of cytomorphological identification of CTC-clusters enriched by filtration [25]. We therefore decided to use ScreenCell^®^ filters to investigate the presence of CTC-clusters in both MBC and EBC patients.

### 2.2. Detection of CTC-Clusters by a Size-Based Approach in Patients with Breast Cancer

#### 2.2.1. Patient Characteristics

Between June 2014 and December 2015 a total of 37 and 23 patients with EBC and MBC undergoing systemic treatment were enrolled in the study. Main clinical and pathological features are reported in Table 2 and Table 3 (for EBC and MBC patients, respectively).

The median age of EBC patients treated with neoadjuvant chemotherapy (NAC) was 49 years (range 26–84). At diagnosis, tumor size was 2–5 cm (cT2) in 20 patients (54%), and >5 cm (cT3-4) in 16 patients (43%). Clinical nodal status was positive (cN1–3) in 29 cases (78%). No patients with stage I BC were enrolled. Histological grade 3 was reported in 22 evaluable patients (60%). Among the 36 evaluable patients, the median Ki67 value was 40%, with values ranging from 10% to 90%. Thirty-two patients (86%) had primary tumors with a Ki67 staining ≥ 20 %. Nine patients (24.3%) reached a pathological complete response (pCR).

The median age of MBC patients was 68 years (range 29–84). The most common histological type was invasive ductal carcinoma (65% of cases). Of the 23 patients included in the study, 6 (26%) had visceral and 12 (52%) had non-visceral involvement. Eight patients (35%) presented with de novo metastases. All patients, except three, had received no prior systemic treatment for metastatic disease.

#### 2.2.2. CTC-Clusters in Patients with Metastatic and Early Breast Cancer

**Figure 2:**
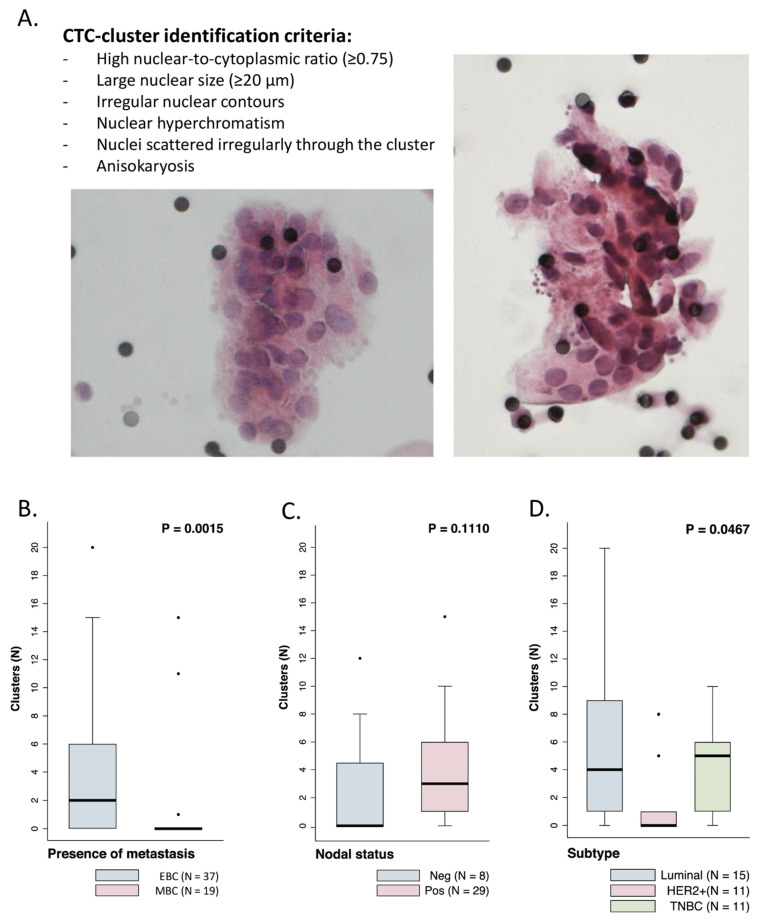
Detection of CTC-clusters in patients with early and metastatic breast cancer. (**A**) Representative images of CTC-clusters enriched by filtration using ScreenCell filters. The list of cytomorphological criteria used for the identification of CTC-clusters is reported in the inset. (**B**–**D**) Boxplots reporting the number of CTC-clusters detected in baseline samples collected from EBC vs. MBC patients (**B**); and in baseline samples collected from EBC patients, according to the patients’ nodal status (**C**) and to the disease subtype (**D**).

**Figure 3:**
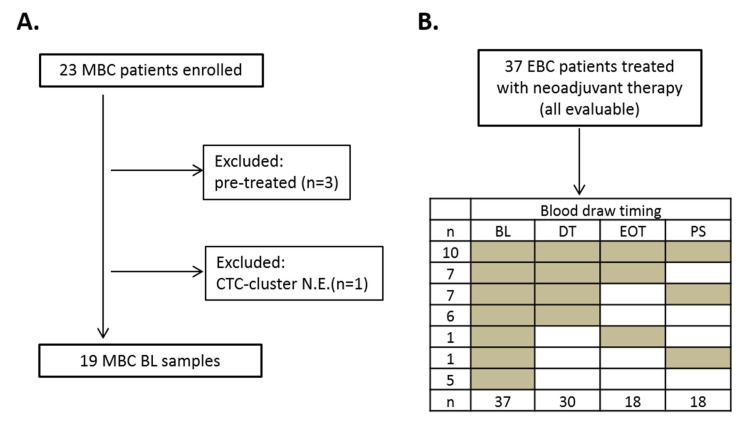
CONSORT plot reporting patients included in the study. (**A**) Metastatic breast cancer (MBC) patients (N.E. = not evaluable: BL = baseline); (**B**) Non metastatic breast cancer (EBC) patients: blood samples available at each time point are reported (BL = baseline; DT = during treatment; EOT = end of treatment; PS = post-surgery).

To investigate the presence of CTC-clusters in our cohort of patients with MBC and EBC, blood samples collected before starting systemic treatment underwent CTC-cluster enrichment by filtration, followed by a marker-independent CTC-cluster identification based on cytomorphological criteria using ScreenCell^®^ filters (Figure 2A). This simplified identification strategy requires only H&E staining rather than immunofluorescence, and it gives reliable results regarding cell malignancy, independently from the expression of specific markers [27,28,29,30], as we have previously confirmed by CTC-cluster genomic profiling [25]. At baseline, in EBC patients, one or more CTC-clusters were detected in 26/37 cases (70%), with a median of two clusters per sample (range 0–20) (Figure 2B). Among the 23 baseline samples collected from MBC patients, three samples were from pre-treated patients and one was not evaluable for CTC-cluster identification (Figure 3A); CTC-clusters were detected in 4 of the 19 remaining samples (21%), with a median of 0 CTC-clusters per sample (range 0–15). CTC-clusters were therefore more frequent and more abundant in patients with EBC than MBC (*p* = 0.0015), despite the fact that notoriously single CTCs are more numerous in MBC.

In particular, patients with stage II BC showed a higher CTC-cluster count than patients with stage III and IV BC (Figure S2A). Among patients with EBC, a slightly higher number of CTC-clusters was detected in patients with node-positive status (Figure 2C), although this difference was not statistically significant (median CTC-cluster number = 0 versus 3 for node-negative versus node-positive patients, *p* = 0.1110). CTC-clusters were more frequently observed in patients with luminal-like and triple negative BC than in patients with HER2-positive disease (median CTC-cluster number = 4, 5, and 0 for luminal-like, triple-negative, and HER2-positive BC respectively, *p* = 0.0467) (Figure 2D). For 25 patients for whom a primary tumor tissue sample was available, the presence of CTC-clusters was analyzed with respect to the presence of tumor-infiltrating lymphocytes (TILs) at the primary tumor site but no difference in CTC-cluster counts was observed between patients presenting a high or low level of TILs (median CTC-cluster number = 3 versus 2 for patients with <12% versus ≥12% TILs, *p* = 0.5392) (Figure S2B).

These results indicate that CTC-clusters are present in early stages in BC patients and are more frequent than in MBC patients. Among EBC patients, CTC-clusters are more abundant in the blood of patients with HER2-negative disease.

#### 2.2.3. Longitudinal Evaluation of CTC-Clusters during Neoadjuvant Therapy

**Figure 4:**
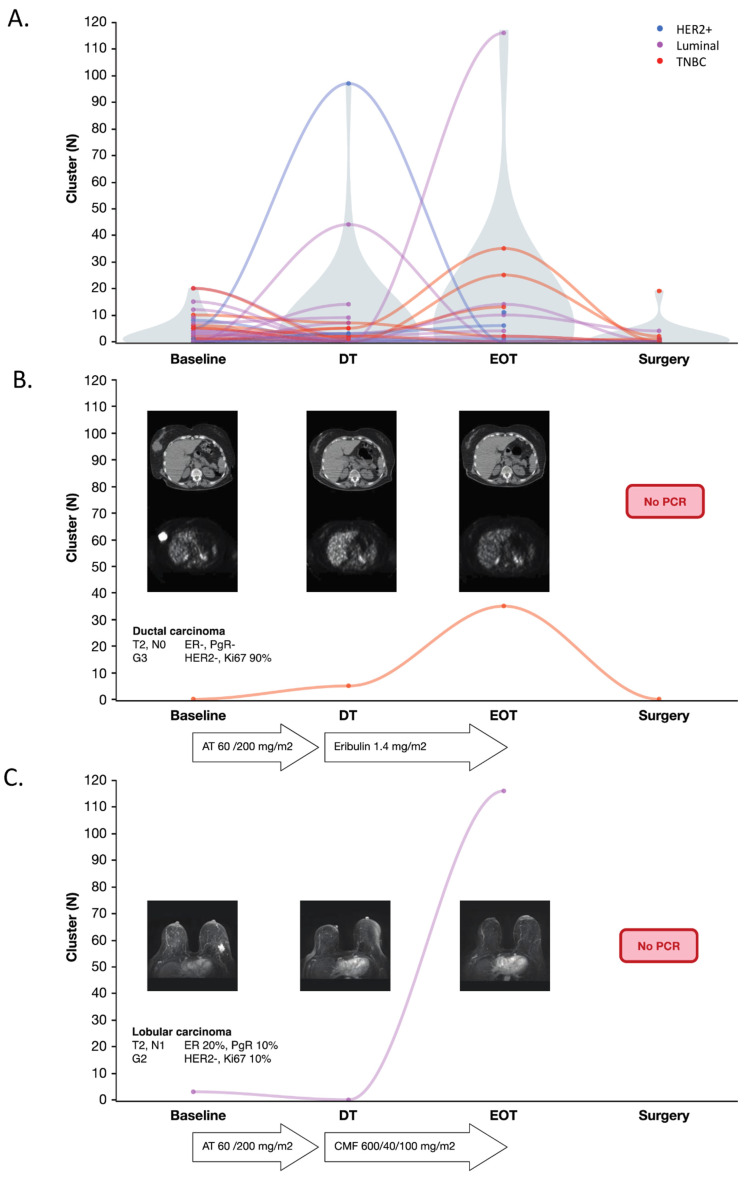
CTC-cluster evaluation during neoadjuvant therapy in early breast cancer patients. (**A**) Violin plot showing the number of CTC-clusters detected in samples longitudinally collected from 37 EBC patients. CTC-clusters were evaluated before starting neoadjuvant treatment (Baseline, *n* = 37), during (DT, *n* = 30), at the end of therapy (EOT, *n* = 18), and after surgery (Surgery, *n* = 18). The colors indicate the BC subtype (blue = HER2-positive; purple = luminal-like; red = triple-negative) while the gray shadow indicates the density of samples for the corresponding CTC-cluster number. The detailed description of 2 index cases is reported in panels (**B**,**C**). TNBC = triple-negative breast cancer; AT = Antracyclines, Taxanes; CMF = Cyclophosphamide, Methotrexate, Fluorouracil; pCR = pathological complete response.

To further investigate the clinical relevance of CTC-clusters in EBC patients, longitudinal blood samples collected at baseline (*N* = 37), during (*N* = 30), at the end (*N* = 18) of NAC and after surgery (*N* = 18) were analyzed (Figure 3B and Figure 4A). The median number of detected CTC-clusters at baseline was 2 (range 0–20), during treatment (DT) was 1 (range 0–97), and at the end of treatment (EOT) was 3 (range 0–116). Thus, CTC-clusters did not decrease during NAC, but instead increased in some patients. Overall, no significant differences were observed in DT and EOT with respect to baseline. On the other hand, a significant decrease was observed from DT to surgery (*p* = 0.0448) and EOT to surgery (*p* = 0.0208). Only a slight decrease was instead observed between baseline and surgery (*p* = 0.0678). The median number of CTC-clusters after surgery was 0 (range 0–20).

At baseline, numerically fewer clusters were observed in NAC-responders, i.e., patients with complete disappearance or a reduction of primary tumor volume of at least 50% after NAC, as compared to non-responders, i.e., patients with stable disease after NAC: 1 cluster (range 0–20) versus 4 clusters (range 0–12), respectively (*p* = 0.58). The presence of CTC-clusters at baseline was not significantly associated with pCR (Figure S3A). However, patients without clusters at baseline reported a numerically higher pCR rate as compared with those presenting with clusters, 27% versus 23%, respectively. Moreover, after surgery, a significantly lower number of clusters was observed in patients with pathological complete or partial response versus stable disease (*p* = 0.0208) (Figure S3B). As of 15 May 2020, a total of 10 out of 37 EBC patients relapsed. No difference in baseline or post treatment distribution of clusters was reported among patients with or without a relapse. At the same date, 4 out of 19 evaluable MBC patients had died, notably the negative predictive value of clusters at baseline in this case was as high as 86%, but the data is merely explorative due to the small sample size.

We present two examples of patients who responded to NAC but did not achieve pCR, illustrating the cluster’s dynamics during treatment.

Patient A (Figure 4B) was diagnosed with a 40 mm ductal carcinoma of the right breast, G3 ER, PgR and HER2 negative, 90% Ki67. Bone scan and liver ultrasound were negative for distant involvement. She was further staged with a positron emission tomography (PET) scan that confirmed a breast primary lesion with a standardized uptake value (SUV) of 22.5 and no loco-regional involvement. The patient was therefore enrolled in a NAC clinical trial and received four cycles of Doxorubicin 60 mg/m^2^ together with Paclitaxel 200 mg/m^2^ q21. No clusters were detectable at baseline. The first PET evaluation showed a dramatic drop in metabolic activity (SUV 3.7), with five clusters detectable in the peripheral blood. Eribulin 1.23 mg/m^2^ was then started and four cycles were administered with a 1, 8, q21 schedule. The subsequent PET scan showed further metabolic response with a 3.7 SUV, while an increase in clusters was observed (35 clusters). She then underwent a quadrantectomy with 17 mm residual disease and absence of nodal involvement (ypT1c, N0). ER was 2%, PgR and HER2 were negative, Ki67 was confirmed at 90%. Filter based enumeration after surgery showed a complete clearance of detectable clusters. The patient was then started on adjuvant CMF (Cyclophosphamide 600 mg/m^2^, methotrexate 40 mg/m^2^ and 5-Fluorouracil 600 mg/m^2^) but died seven months after surgery due to noncancer-related causes without any detectable local or distant relapse.

Patient B (Figure 4C) was diagnosed with a screening-detected lobular carcinoma of the left breast, G2, ER 20%, PgR 10%, HER2 negative and Ki67 10%. The baseline breast magnetic resonance (MRI) showed a multifocal primary tumor with a 38 mm main lesion and a 3 mm satellite lesion, while distant metastases were excluded via PET scan. Baseline clusters enumeration was 3. A Doxorubicin 60 mg/m^2^ and Paclitaxel 200 mg/m^2^ q21 based NAC was started. Breast MRI after 4 cycles showed a partial regression. Clusters were not detectable. CMF was administered for 4 cycles. While the breast MRI showed further radiological response, 116 clusters were detected in the peripheral blood. The patient underwent quadrantectomy with 3 mm residual disease and two metastatic lymph nodes out of seven analyzed (ypT1a N1a). ER was 90%, PgR and HER2 were negative, Ki-67 was 5%. After bone scan restaging, she was started on adjuvant Letrozole 2.5 mg, which is still ongoing without evidence of distant or local recurrence.

## 3. Discussion

By using an epitope-independent enrichment method combined with cytomorphologic detection and picking of single CTC-clusters we have previously reported the presence of genomically aberrant cells within 46/48 CTC-clusters isolated from six early-stage breast cancer patients [25]. In the current study, we extended these findings by providing methodological comparison between enrichments methods and reporting results on CTC-cluster detection comparing EBC with MBC patients. We first challenged the most frequently-used technical approach in BC for CTC-cluster detection, the CellSearch^®^, by comparing it with methods based on size exclusion. Overall, filtration-based methods allowed detecting a higher number of clusters in the blood of BC patients. Thus, we next analyzed blood samples prospectively collected from 37 EBC and 23 MBC patients using the ScreenCell approach and reported that, surprisingly, CTC-clusters were more frequently detected in EBC than in MBC patients. We also observed that molecular subtypes affected their presence in EBC as clusters were more frequently observed in women with HER-2 negative primaries. Finally, the presence of clusters before starting neoadjuvant treatment did not associate with pCR and their numbers increased during treatment, but dropped after surgery.

To the best of our knowledge, this is the first study specifically comparing CTC-cluster detection by CellSearch^®^ and by a validated filtration-based technique [31], in patients with BC. Such a comparison has instead been performed in small-cell lung cancer patients, by using in parallel the CellSearch^®^ and the ISET filtration approach, showing similar results [22]. Indeed, in lung cancer patients, no clusters were detected with the CellSearch^®^, whereas they were found in 50% of samples from stage IIIB/IV patients processed with the ISET. The findings were explained by the authors as a possible failure of the immune-magnetic enrichment step in the CellSearch^®^ protocol to capture large size clusters. However, additional considerations can be made regarding the increased CTC-cluster detection attained by using filtration, both in our study and in that by Krebs and colleagues. Strong positivity for mesenchymal, with concomitant weak positivity for epithelial markers, has been reported for CTC-clusters isolated from patients with advanced breast cancer [24]. Thus, an increase in cluster detection is not surprising when using methods that do not relay on the expression of epithelial markers, and which are not limited to the detection of epithelial clusters only. Conversely, the observed increased detection of epithelial clusters (CK^pos^) is an unexpected finding. A possible explanation is that CK^pos^ CTC-clusters can also include cells undergoing EMT and therefore expressing a mixed phenotype rather than a frankly epithelial one. Since the CellSearch^®^ detects only CTC-clusters expressing both EpCAM and CK, but EpCAM expression is lost early during EMT [23,32], the CellSearch^®^ could miss CK^pos^ CTC-clusters that are undergoing EMT. This hypothesis could not be tested in the present study since the expression of EpCAM and mesenchymal markers was not assessed and this represents a limitation. However, the results of the spiking experiments showing that CellSearch^®^ yielded comparable recovery rates as filtration devices, when using frankly epithelial mammospheres (expressing both EpCAM and CK) indirectly supports our hypothesis. Thus, since in the numerous studies run with the CellSearch^®^ in women with early disease, massive presence of clusters has not been reported, we speculate that the higher detection frequency of CTC-clusters in EBCversus MBC patients observed here using filtration is related to a more mesenchymal or mixed phenotype of CTC-clusters specifically in the early stages of BC. Further investigations comparing epitope-independent approaches to the CellSearch^®^ in different BC stages will be required to address this question.

Besides filters, other marker-independent technologies such as the ^HB^CTC-Chip [4,24], the Cluster-Chip [33] and the Parsortix™ [6], have been employed for CTC-cluster studies, but mainly focusing on functional aspects rather than on pure translational purposes. In fact, despite the fact that a number of studies have described new technical tools specifically dedicated to CTC-cluster detection [for a review see [34,35], none of these innovative methods is widely available to clinical research centers. In this context, simpler technologies, as those based on size-exclusion would represent an affordable approach, easily transferable to clinical studies that might help in elucidating the role of CTC-clusters in different clinical contexts. This represents a strength of our study and opens the way to further investigations on the role of CTC-clusters in larger series of women with EBC.

Indeed, here we applied an easy-to-use filtration-based approach to investigate the relevance of CTC-clusters in 37 EBC and in 23 MBC patients. The ScreenCell^®^ technology was chosen since its validity has already been reported both for single CTCs and for clusters both when identified based on cytomorphological criteria only, [29,30] or based on marker expression [36].

Overall we report that, in baseline samples collected at the beginning of NAC, the detection of at least 1 CTC-cluster occurred at least three times more frequently in women with early breast cancer than in women beginning first line treatment for MBC (a result that we also observed in our previous pilot study, which was comparing ScreenCell^®^ with AdnaTest technology [37]). Although, due to the small case series, we have not done a formal analysis to exclude a bias due to different distribution of molecular subtypes between the two groups, molecular subtype linked effects would have impacted the data in opposite direction than observed. Thus, our findings support the concept that dissemination of CTC-clusters is an early event in EBC patients, rather than an event occurring during metastatic progression, as might have been expected by the high metastatic potential of clusters [4]. Since dissemination is proven to occur early in breast cancer [8,9], and indeed single CTCs also hold prognostic value in EBC women [11,12,13], the more frequent presence of CTC-clusters and the higher number of clusters seen in early rather than later steps of the disease is intriguing.

Nevertheless, many questions on clinical and biological aspects still remain to be answered. We observed that molecular subtypes affect the prevalence of CTC-clusters. In particular, CTC-clusters were found to be significantly more frequent in women bearing HER2-negative tumors, a result that may appear counterintuitive since HER2-positive tumors are more aggressive and are frequently associated with stemness markers [38]. Moreover, we have noticed that luminal-like tumors release high number of clusters, a finding possibly linked to their late relapse-pattern and to a more efficient promotion of dormancy within the clusters from patients with ER+ tumors [39]. Overall, this suggests that clusters should be studied in molecularly homogeneous populations, although this could not be done in this study, due to the limited number of patients.

In our cohort of EBC patients, the detection of clusters did not correlate with the likelihood of achieving pCR, a finding already reported in the literature for CTCs [12,30]. Moreover, during the course of treatment a trend towards an increase in CTC-clusters rather than a decrease was observed, as also described in another study using ScreenCell^®^ filters [30]. Indeed, only after surgery did we actually observe a decrease in the number of clusters, although a significantly higher number of clusters persisting after surgery was detected in patients with a pathologically non-responding disease (median 3, IQR 1–11.5 vs. 0, IQR 0–1 for non-responders and responders, respectively).

Thus, it may be speculated that in EBC, clusters formation is related to the presence and characteristics of primary tumor, and the neoadjuvant treatment has a different effect on the primary tumor and on clusters. Moreover, despite the fact that this study is not properly powered to detect differences in disease-free survival and no association was observed between relapse and CTC-clusters at baseline, it is intriguing to think of potential applications of cluster enumeration after surgery as a completion of pathological staging to assess the overall combined response to systemic and locoregional treatments.

Notably, a discrepancy between cluster dynamics and imaging was observed. As consistently shown by the index cases, clusters generally increased during NAC, notwithstanding the concomitant radiological and metabolic response. On the other hand, patients that did not show response to NAC had a significantly higher number of clusters after surgery. This suggests a more nuanced role of clusters in EBC with respect to that of epithelial clusters in the metastatic setting.

Regarding the cluster composition, we reported in the same issue of the journal [25] that most CTC-clusters isolated in women with EBC are heterotypic with variable proportions of tumor and accessory normal cells. Indeed, cooperation and crosstalk with other blood cells play a relevant role in increasing the metastasis-promoting efficiency of cluster [7,40,41,42,43]. However, in the current study, we did not find an association between TILs evaluated on the primary tumor and CTC-clusters, and thus the possible interaction between inflammatory cells and CTC-clusters warrants further studies.

Unfortunately we were instead unable to perform a genomic profiling of the clusters detected in the present case series similarly to what was done in the our other study due to the fact that here clusters were collected from the filters months after their isolation resulting in bad-quality amplified DNA not suitable for sequencing. Nonetheless, our previous data [25] confirmed the malignancy of 96% of clusters identified by filtration and cytomorphologic evaluation in EBC patients, supporting the validity of our detection approach.

The observation that clusters do not disappear with the neoadjuvant treatment (and thus possibly also persist after adjuvant treatment) suggests that they might hold the potential of biomarkers of response to monitor the efficacy of neo- and adjuvant treatments, although this awaits a demonstration in larger studies. Furthermore, once their role in the metastatic process will be widely confirmed, they could also become the target of specific treatments as already done in the metastatic setting (NCT03928210) where evidences on the prometastatic role of CTC-clusters are available [6,44].

We are finally aware of the limits due to the small size and heterogeneity of the case series, although its strength may be linked to the fact that these represent real-world patients, prospectively collected within the daily clinical practice.

## 4. Materials and Methods

### 4.1. Cell Cultures and Spiking Experiments

The MCF7 breast cancer cell line was purchased from the American Type Culture Collection (ATCC, Manassas, VA, USA) and cultured in DMEM/F-12 (Lonza, Slough, UK) medium supplemented with 10% fetal bovine serum (Lonza). Mammospheres were derived from MCF7 cells cultured as floating cells in MammoCult™ (StemCellTechnologies, Vancouver, BC, Canada), a serum-free culture medium, supplemented with Heparin Solution (StemCell Technologies) at final concentration of 4 µg/mL, and Hydrocortisone (StemCell Technologies) at final concentration of 0.48 µg/mL. The cells were maintained in non-adherent condition (Corning^®^ Ultra-Low Attachment flask, Corning Inc., Corning, NY, USA) at 37 °C, in humidified 5% CO_2_ and 5% O_2_. Authentication of cell lines by STR DNA profiling analysis was performed by the Genomic Core Facility at Fondazione IRCCS Istituto Nazionale Tumori (INT). We adopt a *Mycoplasma* contamination testing policy employing an ELISA approach (MycoAlert mycoplasma detection kit, Lonza) for regular testing. All cells used for this study tested negative for mycoplasma.

For the spiking experiments, either single MCF7 cells or single mammospheres were manually captured under an inverted microscope using a p10 micropipette and directly spiked into phosphate-buffered saline (PBS) supplemented with human serum albumin (HSA 3% *w/v*, to mimic protein concentration of plasma), or into healthy donor blood collected in either CellSave Preservative Tubes (Menarini Silicon Biosystems, Bologna, Italy) for CellSieve™ and CellSearch^®^ processing, or in K_2_EDTA BD Vacutainer tubes (BD, Franklin Lakes, NJ, USA) for ScreenCell^®^ processing. Spiked-in samples were processed following the same protocols used for clinical samples, described in paragraphs 4.2 and 4.5. Spiked-in mammospheres were variable in dimensions. We have chosen spheres that had sizes similar to those of medium/large clusters observed in patients, since the main reason for these experiments was to exclude possible shearing stress effect occurring during filtration which could damage the clusters (which should have a major impact on large rather than small clusters).

### 4.2. Comparison of CellSearch^®^ and CellSieve™ Filters for CTC-Cluster Detection in Clinical Samples

Peripheral blood samples (15 mL) were collected in CellSave Preservative Tubes (Menarini Silicon Biosystems) from patients with MBC treated at the Robert H Lurie Comprehensive Cancer Center at the Northwestern University (Chicago, IL, USA). All patients provided written informed consent to participate in the study, which was approved by the institutional review board at the Robert H. Lurie Comprehensive Cancer Center of Northwestern University (NUDB16Z01). Each sample was divided into two aliquots (7.5 mL each) and processed in parallel with the CellSearch^®^ (Menarini Silicon Biosystems) and with CellSieve™ filters (Creatv MicroTech, Potomac, MD, USA) within one day of collection. For CellSearch^®^ processing, the CELLSEARCH^®^ Circulating Tumor Cell Kit (Menarini Silicon Biosystems) was used following the manufacturer’s instructions. Briefly, after immunomagnetic enrichment based on EpCAM expression, enriched CTCs were stained with fluorescently-labeled antibodies against cytokeratins (CK) (8, 18 and 19) and CD45 and with DAPI. The number of CTC-clusters (groups of ≥2 CK^pos^/CD45^neg^ cells) was evaluated using the CELLTRACKS ANALYZER II^®^ System (Menarini Silicon Biosystems) by a trained technician. For CellSieve™ filters processing, the CellSieve™ Enumeration Kit (Creatv MicroTech, Rockville, MD 20850, USA) was used following the manufacturer’s instructions. The blood samples were filtered through a microporous membrane with pores of 7 µm diameter and subsequently stained with fluorescently-labeled antibodies against CK (8, 18 and 19), CD45 and CD31 (an endothelial marker used to exclude endothelial cell clusters) and with DAPI. The number of CTC-clusters (i.e., groups of ≥2 CK^pos^/CD45^neg^ or ≥2 CK^neg^/CD45^neg^/CD31^neg^ cells for CK^pos^ and CK^neg^ CTC-clusters, respectively) was evaluated using a fluorescence microscope.

### 4.3. Case Series & Blood Sample Collection Timing

Women with a histologically confirmed diagnosis of stage II and III BC (EBC) were recruited at Fondazione IRCCS Istituto Nazionale dei Tumori (Milan, Italy) prior to start of neoadjuvant treatment as for clinical practice, whereas women with stage IV BC (MBC) were recruited prior to start of the first line of treatment. All patients provided written informed consent before undergoing any procedures and the CTC study was approved by the INT Institutional Review Board and Ethics Committee on February 19 2013.

Blood samples were longitudinally collected from patients with EBC (i) before starting neoadjuvant treatment, (ii) at midcourse during treatment, (iii) at the end of treatment, and (iv) after surgery (from 3 to 27 weeks). Blood samples were collected from patients with MBC before starting the first line of treatment (Figure S2).

Pathological complete response (pCR) was defined as the absence of cancer cells in the surgical specimens of breast and lymph nodes. Partial response (PR) and stable disease (SD) referred to clinical assessment of response to treatment according to the WHO criteria, hence a >50% tumor shrinkage occurred for a PR, and >25% tumor increase for progressive disease (PD), whereas stable disease was neither PR nor PD.

### 4.4. Patho-Biological Characterization of Tumors

Hormone receptor status was evaluated according to the American Society of Clinical Oncology guidelines [45]. HER2 status was considered negative when the immune-histochemical score was 0–1, or 2+ with a negative chromogenic in situ hybridization result [46]. Ki-67 labeling index was assessed by the MIB-1 monoclonal antibody by counting invasive cancer cells at the tumor periphery, without focusing on hot-spots, as recommended by the International Ki-67 in Breast Cancer Working Group [47].

The evaluation of tumor-infiltrating lymphocytes (TILs) was performed in full-face hematoxylin and eosin sections from surgical or bioptic sample, strictly adhering to the criteria proposed by the TILs Working Group [48]. Briefly, all mononuclear cells (i.e., lymphocytes and plasma cells) in the stromal compartment within the borders of the invasive tumor were evaluated and reported as a percentage. TILs outside the tumor border, around in situ component (DCIS) and normal breast tissue, as well as in areas of necrosis, were excluded from the scoring.

### 4.5. CTC-Cluster Enumeration by ScreenCell^®^ Filters

Peripheral blood samples (9 mL), collected into K_2_EDTA BD Vacutainer tubes (BD) using a 21G needle, were stored at 4 °C in the dark and processed within 2.5 h for CTC-cluster enrichment using the ScreenCell^®^ Cyto kit (ScreenCell, Sarcelles, France) [49] according to the manufacturer’s instructions, with slight modifications with respect to what previously described [37,50]. Briefly, after discarding the first aliquot of blood to avoid contamination by keratinocytes, three aliquots of 3.0 mL of whole blood per sample were separately mixed with 4 mL of a proprietary red blood cell lysis and fixation buffer (ScreenCell^®^ FC2 filtration buffer) and incubated for 8 min at room temperature. Each aliquot was filtered to isolate CTC-clusters using ScreenCell^®^ Cyto isolation supports (ISs), consisting in a microporous membrane with pores of 6.5 µm diameter. After rinsing with PBS, ISs were air-dried and stained with Hematoxyilin Solution S (Merck, Darmstadt, Germany) for 1 min and Shandon Eosin Y Aqueous Solution (Thermo Fisher Scientific Inc., Waltham, MA, USA) for 30 s, at room temperature; or with May Grünwald (Merck Millipore, Burlington, MA, USA; incubation for 2.5 min followed by a second incubation for 2.5 min in May Grünwald diluted 1:2 with water) and Giemsa (Merck Millipore; diluted 1:10 with water, 10 min incubation) at room temperature. The stained ISs were sent to ScreenCell for evaluation by a certified pathologist according to published criteria [51]. CTC-clusters were defined as clusters of ≥2 CTCs showing the criteria of malignancy: nuclear size ≥20 μm, nuclear-to-cytoplasmic ratio ≥0.75, irregular nuclear contours and nuclear hyperchromatism. In case the cytoplasm edges were not clearly visible inside the cluster (preventing nuclear-to-cytoplasmic ratio evaluation), malignancy identification was mainly based on nuclei appearance: nuclei scattered irregularly through the cluster and anisokaryosis (i.e., nuclei of variable sizes and shapes), in addition to nuclear size ≥20 μm and irregular nuclear membrane. Detailed guidelines for ScreenCell filter interpretation are described elsewhere [51]. Samples showing poor quality of cytology were excluded from the analysis. The total number of CTC-clusters for each sample was obtained by summing the CTC-clusters identified in the 3 ISs (corresponding to 9 mL of blood).

### 4.6. Statistical Analysis

Clinical and pathological variables were reported through descriptive analyses. Categorical variables were reported as frequency distribution, whereas continuous variables were described according to median and interquartile range (IQR). Differences in clusters distribution across subgroups of interest were tested through Mann–Whitney U test. Pairwise comparison between CellSearch^®^ and CellSieve™ technologies, and across different time points during neoadjuvant therapy were performed though Wilcoxon sign-rank test. All reported *p*-values are two-sided.

Statistical analysis was conducted using the StataCorp 2016 Stata Statistical Software: Release 15.1 (College Station, TX, USA), R (The R foundation for Statistical Computing. version 3.3.1) (21 June 2016) and JMP (SAS Institute, version 15).

## 5. Conclusions

This study represents a small snapshot of CTC-cluster detection methods and on the prevalence of clusters in BC patients at different disease stages. Nonetheless, it highlights the possible bias linked to inadequate methods for cluster detection, a technical bias that is worth considering in future translational studies. In addition, we report a new observation of the fact that CTC-clusters are frequent in women with EBC. This represents a provocative finding that needs to be addressed in future studies on larger series of cancer patients, homogeneous with respect to molecular subtype. Finally, the observation that CTC-clusters do not disappear during neoadjuvant treatment fosters the importance of developing treatments specifically aimed at interfering with them.

## Figures and Tables

**Table 1:** Mammosphere recovery in spiking experiments, using different detection methods.

Sample ID	Enrichment Technology	Mammosphere Recovery Rate (%)
1	CellSearch^®^	70
2		80
3		60
4	CellSieve™	70
5		80
6		60
7	ScreenCell^®^	60
8		100
9 *		100
10 *		100

* For samples 9 and 10, mammospheres were spiked into PBS supplemented with HSA, instead of blood.

**Table 2:** Clinico-pathological characteristics of EBC patients and CTC-clusters.

	*N*	%	Median CTC-Clusters (Range)	*p*	CTC-Cluster + (%)	*p*
**Age**						
• <50	20	54.1	2.5 (0–20)	0.889	15 (75%)	0.719
• ≥50	17	45.9	2 (0–20)		11 (65%)	
**Tumor size**						
• T1–T2	21	56.8	4 (0–20)	0.180	16 (76%)	0.475
• ≥T3	16	43.2	1 (0–15)		10 (63%)	
**Nodal status**						
• N0	8	21.6	0 (0–12)	0.273	3 (37.5)	0.123
• N1	21	56.8	3 (0–20)		17 (81.1)	
• ≥N2	8	21.6	3 (0–20)		6 (75%)	
**Histology**						
• NST	35	94.6	2 (0–20)	0.322	15(68%)	
• Lobular	2	5.4	3 (0–15)		11(73%)	>0.99
**Grade**						
• 2	10	27.0	2 (0–15)	0.918	7 (70%)	>0.99
• 3	22	59.5	1.5 (0–20)		15 (68.2%)	
• Missing	5	13.5				
**Ki67**						
• <20	4	10.8	1.5 (0–12)	>0.10	2 (50%)	0.570
• ≥20	32	86.5	2 (0–20)		23 (72%)	
• Missing	1	2.7	-		-	
**Subtype**						
• HER2-positive	11	29.7	0 (0–8)	0.047	5 (45%)	0.111
• Triple negative	11	29.7	5 (0–20)		9 (82%)	
• Luminal-like	15	40.5	4 (0–20)		12 (80%)	
**Type of neoadjuvant chemotherapy**						
• Anthra/Taxane	32	86.5	2.5 (0–20)	0.984	22 (69%)	0.609
• CarboPt-based	5	13.5	1 (0–20)		4 (80%)	

**Table 3:** Clinico-pathological characteristics of MBC patients.

	*N*	%
**Age**		
• <50	5	21.7
• ≥50	18	78.3
**Histology**		
• Ductal	15	65.2
• Lobular	2	8.7
• Other	6	26.1
**Disease type at screening**		
• Visceral	6	26.1
• Nonvisceral	12	52.2
• Missing	3	13.0
**Hormone receptor status**		
• ER -positive, PgR positive or both	18	78.3
• ER-negative and PgR-negative	5	21.7
**HER2 status**		
• Positive	1	4.3
• Negative	22	95.7
**Metastatic disease at diagnosis**		
• No	15	65.2
• Yes	8	34.8
**Prior chemotherapy for metastatic disease**		
• No	20	87.0
• Yes	3	13.0

## Data Availability

The datasets used and/or analysed during the current study are available from the corresponding author on reasonable request.

## Supplementary Materials

The following are available online at https://www.mdpi.com/article/10.3390/cancers13102356/s1, Table S1: Clinico-pathological characteristics of the patients’ cohort for technological comparison, Table S2: CTC-cluster count in samples processed in parallel with CellSearch and CellSieve filters, Figure S1: CTC-clusters expressing CD31, Figure S2: Association of CTC-clusters with clinic-pathological characteristics in patients with EBC, Figure S3: Association of CTC-clusters with outcome in EBC patients.

## Abbreviations

BC: breast cancer; CK: cytokeratin; CTC: circulating tumor cell; DT: during treatment; EMT: epithelial-to-mesenchymal transition; EOT: end of treatment; HSA: human serum albumin; IQR: interquartile range; IS: isolation support; MBC: metastatic breast cancer; MRI: magnetic resonance imaging; NAC: neoadjuvant chemotherapy; EBC: early breast cancer; OS: overall survival; pCR: pathological complete response; PBS: phosphate-buffered saline; PD: progressive disease; PET: positron emission tomography; PFS: progression free survival; PR: partial response; SD: stable disease; SUV: standardized uptake value; TIL: tumor-infiltrating lymphocytes.
