# Supplementary material for: Circulating Tumor Cell Clusters Are Frequently Detected in Women with Early-Stage Breast Cancer
Source: Cancers (Basel). 2021 May 13;13(10):2356. doi: 10.3390/cancers13102356 (PMC8153325; doi:10.3390/cancers13102356)
Supplement: Supplementary file 1 [file cancers-13-02356-s001.zip › cancers-1111031-supplementary.pdf]

cancers

Supplementary Materials

Circulating Tumor Cell Clusters are Frequently Detected in Women with Early-Stage Breast Cancer

Carolina Reduzzi [1,2,†], Serena Di Cosimo [1,†], Lorenzo Gerratana [2,3,4], Rosita Motta [1], Antonia Martinetti [5], Andrea Vingiani [6], Paolo D'Amico [2,7], Youbin Zhang [2], Marta Vismara [1], Catherine Depretto [8], Gianfranco Scaperrotta [8], Secondo Folli [9], Giancarlo Pruneri [6], Massimo Cristofanilli [2], Maria Grazia Daidone [1] and Vera Cappelletti [1,*]

[1] Department of Applied Research and Technological Development, Fondazione IRCCS Istituto Nazionale dei Tumori di Milano, 20133 Milan, Italy; carolina.reduzzi@northwestern.edu (C.R.); serena.dicosimo@istitutotumori.mi.it (S.D.C.); rosita.motta@istitutotumori.mi.it (R.M.); marta.vismara@istitutotumori.mi.it (M.V.); mariagrazia.daidone@istitutotumori.mi.it (M.G.D.)
[2] Department of Medicine-Hematology and Oncology, Robert H. Lurie Comprehensive Cancer Center, Feinberg School of Medicine, Northwestern University, Chicago, IL, 60611 USA; lorenzo.gerratana@northwestern.edu (L.G.); paolo.damico@northwestern.edu (P.D.); youbin.zhang1@northwestern.edu (Y.Z.); Massimo.cristofanilli@nm.org (M.C.)
[3] Department of Medicine (DAME), University of Udine, 33100 Udine, Italy
[4] Department of Medical Oncology, Centro di Riferimento Oncologico di Aviano (CRO) IRCCS, 33081 Aviano, Italy
[5] Medical Oncology Unit, Fondazione IRCCS Istituto Nazionale dei Tumori, 20133 Milan, Italy; Antonia.Martinetti@istitutotumori.mi.it
[6] Pathology Department, School of Medicine, Fondazione IRCCS Istituto Nazionale Tumori and University of Milan, 20133 Milan, Italy; Andrea.Vingiani@istitutotumori.mi.it (A.V.); Giancarlo.Pruneri@istitutotumori.mi.it (G.P.)
[7] New Drugs and Early Drug Development for Innovative Therapies Division, IEO, European Institute of Oncology IRCCS, 20133 Milan, Italy
[8] Department of Radiology, Fondazione IRCCS Istituto Nazionale dei Tumori di Milano, 20133 Milan, Italy; Catherine.Depretto@istitutotumori.mi.it (C.D.); Gianfranco.Scaperrotta@istitutotumori.mi.it (G.S.)
[9] Breast Cancer Unit, Fondazione IRCCS Istituto Nazionale dei Tumori di Milano, 20133 Milan, Italy; Secondo.Folli@istitutotumori.mi.it
* Correspondence: vera.cappelletti@istitutotumori.mi.it; Tel.: +39-022390-2700
† Equally contributed.

Citation: Reduzzi, C.; Cosimo, S.D.; Gerratana, L.; Motta, R.; Martinetti, A.; Vingiani, A.; D'Amico, P.; Zhang, Y.; Vismara, M.; Depretto, C.; et al. Circulating Tumor Cell Clusters are Frequently Detected in Women with Early-Stage Breast Cancer. *Cancers* **2021**, *13*, 2356. https://doi.org/10.3390/cancers13102356

Academic Editor: Antonio Russo

Received: 29 January 2021
Accepted: 10 May 2021
Published: 13 May 2021

Publisher's Note: MDPI stays neutral with regard to jurisdictional claims in published maps and institutional affiliations.

Copyright: © 2021 by the authors. Submitted for possible open access publication under the terms and conditions of the Creative Commons At-tribution (CC BY) license (https://crea-tivecommons.org/licenses/by/4.0/).

Table S1. Clinico-pathological characteristics of the patients' cohort for technological comparison.

	N	%
	**Histology**	
• Ductal	10	62.5
• Lobular	6	37.5
	**Disease type at screening**	
• Visceral	12	75.0
• Nonvisceral	4	25.0
	**Hormone receptor status**	
• ER -positive, PgR positive or both	11	68.8
• ER-negative and PgR-negative	5	31.2
	**HER2 status**	
• Positive	1	6.2
• Negative	15	93.8

Table S2. CTC-cluster count in samples processed in parallel with CellSearch and CellSieve filters.

**Sample ID**	**CellSearch**	**CellSieve**	**CellSieve**
	**CK^{pos}**	**CK^{pos}**	**CK^{neg}**
1	0	8	6
2	1	4	5
3	1	3	5
4	2	6	5
5	108	112	NE
6	0	5	2
7	0	5	3
8	7	0	1
9	1	2	3
10	0	0	0
11	2	0	0
12	0	1	1
13	0	1	0
14	0	1	0
15	0	0	0
16	2	6	2
17	0	1	2
18	8	15	0
19	25	24	1

NE = Not evaluable due to the high number of cells on the filter.

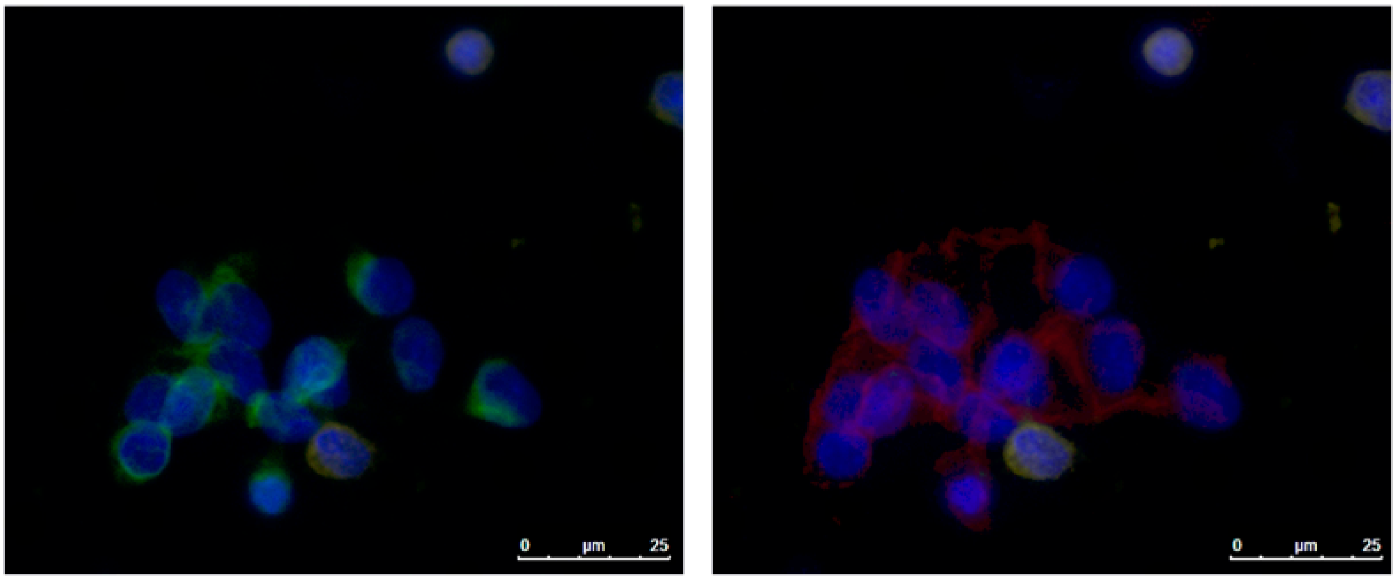

Figure S1. CTC-clusters expressing CD31. Representative image of a CTC-cluster showing expression of both CK (left panel; green = CK, blue = DAPI) and CD31 (right panel; red = CD31). Apart from one blood cell expressing CD45 (in yellow), the rest of the cells forming the cluster are CD45^{neg}.

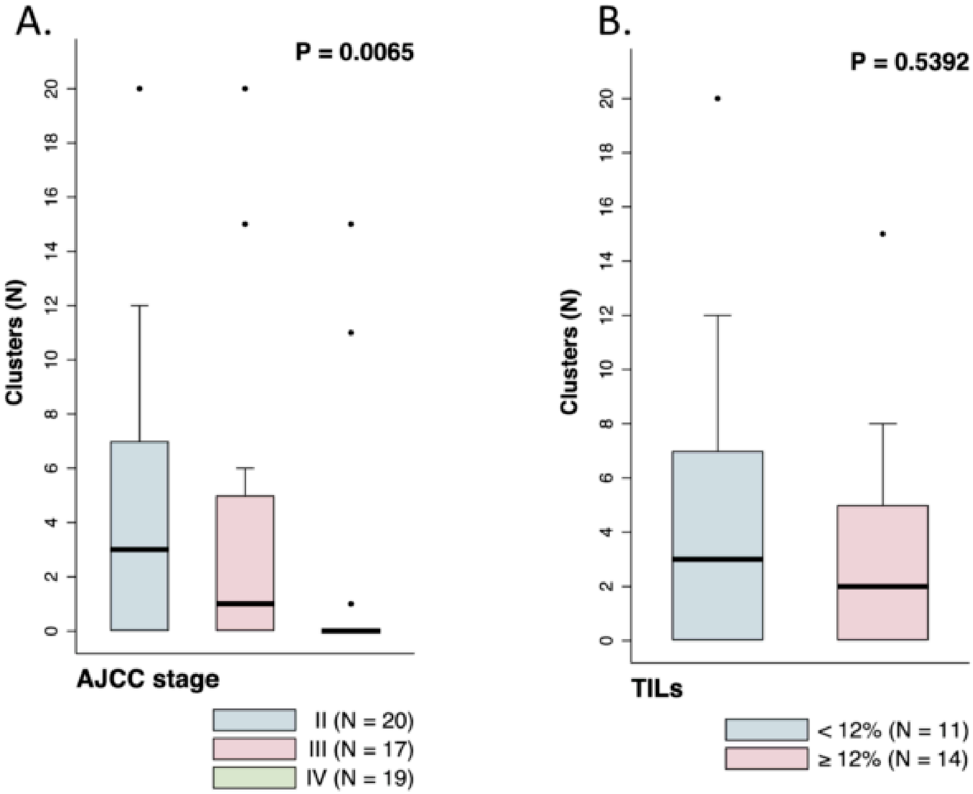

Figure S2. Association of CTC-clusters with clinico-pathological characteristics in patients with EBC. Boxplots reporting the number of CTC-clusters detected in baseline samples, according to patients' AJCC stage (**A**) and to tumor infiltrating lymphocytes (TILs) rates (low = <12%; high = ≥12%) evaluated at the primary tumor site (**B**).

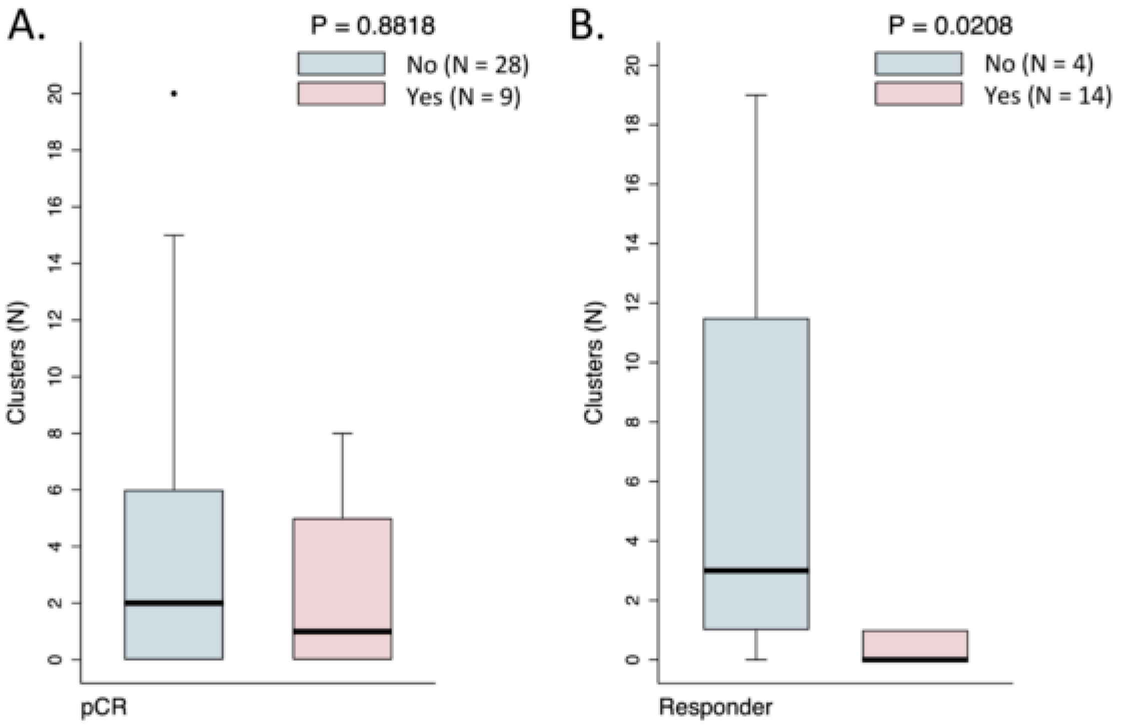

Figure S3. Association of CTC-clusters with outcome in EBC patients. Boxplots reporting the number of CTC-clusters detected (A) in samples collected at baseline, according to the pathological complete response (pCR) evaluation ('no' = patients who did not reach pCR, light-blue bar; 'yes' = patients who reached pCR, violet bar); and (B) in samples collected after surgery, in responder versus non-responder patients ('no' = patients not achieving down-staging, light-blue bar; 'yes' = patients where a down-staging was achieved after NAC, violet bar). A higher post-surgery number of clusters was detectable in patients with no tumor shrinkage after primary chemotherapy.
